# Functional Outcome Prediction in Ischemic Stroke: A Comparison of Machine Learning Algorithms and Regression Models

**DOI:** 10.3389/fneur.2020.00889

**Published:** 2020-08-25

**Authors:** Shakiru A. Alaka, Bijoy K. Menon, Anita Brobbey, Tyler Williamson, Mayank Goyal, Andrew M. Demchuk, Michael D. Hill, Tolulope T. Sajobi

**Affiliations:** ^1^Department of Community Health Sciences, O'Brien Institute for Public Health, University of Calgary, Calgary, AB, Canada; ^2^Department of Clinical Neurosciences, Hotchkiss Brain Institute, University of Calgary, Calgary, AB, Canada; ^3^Department of Radiology, University of Calgary, Calgary, AB, Canada

**Keywords:** machine learning, acute ischemic stroke, functional outcome, clinical risk prediction, discrimination calibration

## Abstract

**Background and Purpose:** Stroke-related functional risk scores are used to predict patients' functional outcomes following a stroke event. We evaluate the predictive accuracy of machine-learning algorithms for predicting functional outcomes in acute ischemic stroke patients after endovascular treatment.

**Methods:** Data were from the Precise and Rapid Assessment of Collaterals with Multi-phase CT Angiography (PROVE-IT), an observational study of 614 ischemic stroke patients. Regression and machine learning models, including random forest (RF), classification and regression tree (CART), C5.0 decision tree (DT), support vector machine (SVM), adaptive boost machine (ABM), least absolute shrinkage and selection operator (LASSO) logistic regression, and logistic regression models were used to train and predict the 90-day functional impairment risk, which is measured by the modified Rankin scale (mRS) score > 2. The models were internally validated using split-sample cross-validation and externally validated in the INTERRSeCT cohort study. The accuracy of these models was evaluated using the area under the receiver operating characteristic curve (AUC), Matthews Correlation Coefficient (MCC), and Brier score.

**Results:** Of the 614 patients included in the training data, 249 (40.5%) had 90-day functional impairment (i.e., mRS > 2). The median and interquartile range (IQR) of age and baseline NIHSS scores were 77 years (IQR = 69–83) and 17 (IQR = 11–22), respectively. Both logistic regression and machine learning models had comparable predictive accuracy when validated internally (AUC range = [0.65–0.72]; MCC range = [0.29–0.42]) and externally (AUC range = [0.66–0.71]; MCC range = [0.34–0.42]).

**Conclusions:** Machine learning algorithms and logistic regression had comparable predictive accuracy for predicting stroke-related functional impairment in stroke patients.

## Introduction

Prognostic risk scores that use patient characteristics to predict functional outcomes in stroke patients are of increasing importance for aiding clinical decisions in stroke management (1). Examples of these prognostic tools include Ischemic Stroke Predictive Risk Score (ISCORE) (2), the Acute Stroke Registry, and Analysis of Lausanne (ASTRAL) (3) and Dense Artery, mRS, Age, Glucose, Onset-to-Treatment, and NIHSS (DRAGON) (4), among others. These models combine multiple predictors to provide insight into the relative or absolute risk of functional impairment for each patient and a simple risk scoring system that allows for their use in busy clinical settings (5–8). These scores are particularly of interest in both routine clinical practice and policy administration for discharge planning, quality improvement, management of prognostic expectations in stroke patients, and resource allocation (9).

One characteristic feature of these prognostic risk scores is that they are mostly developed based on regression models and have shown moderate to good discriminatory accuracy (AUC range = [66 and 88%]) for predicting 90-day functional outcomes in ischemic stroke patients (10). However, these risk scores are inherently limited to a number of reasons. First, existing scores are mostly developed on a highly selective population obtained from randomized controlled trials, which are not representative of the population of stroke patients being seen in acute care settings. Second, the risk scores are mostly developed using a small set of clinical predictors, ignoring the available rich information on patients' clinical, imaging, and behavioral characteristics that may be predictive of the outcome of interest. Third, these risk scores are rarely validated in other external cohorts; they tend to demonstrate poor predictive accuracy even when validated in external cohorts.

Machine learning (ML) algorithms constitute a promising class of methods for developing prognostic models. In recent times, there has been an increased focus on ML algorithms and their potential to revolutionize clinical research, especially in precision medicine. ML algorithms explore both linear and non-linear interactions among predictors while maximizing the information in them to improve the accuracy of outcome predictions. Despite its attractive features and touted potentials, there is still limited uptake of ML for developing prognostic risk scores for stroke patients (10).

In this study, we examine the predictive performance of ML algorithms for predicting a 90-day functional impairment risk after acute ischemic stroke. We hypothesized that the predictive performance of ML would be comparable to the regression-based risk prediction models.

## Methods

The study is reported according to the Transparent Reporting of a multivariable prediction model for Individual Prognosis Or Diagnosis (TRIPOD) checklist for prediction model development and validation (11).

### Data Sources

This study used prospectively collected multicenter observational studies of ischemic stroke patients to develop and validate the ML algorithms for predicting the patient-specific risk of functional impairment. These data sources are described as follows, and the ethics approval were sought from the University of Calgary Conjoint Health Research Ethics Board (REB14-2012 and REB14-2015).

### Precise and Rapid Assessment of Collaterals With Multi-Phase CT Angiography (PROVE-IT) (12)

PROVE-IT is a prospective multi-center hospital-based cohort study of 614 patients with acute ischemic stroke presenting within 12 h of stroke symptom onset with evidence of intracranial occlusion on routine computed tomography angiography CTA and treated with intravenous alteplase and/or intra-arterial therapy. Patients underwent baseline unenhanced CT, multiphase CT angiography, and perfusion CT. Both demographic, clinical, and imaging data were collected on study participants across these centers. The primary outcome was patients' 90-day functional status measured by the mRS. The study was conducted over a 3-year period. Details about this study have been published elsewhere (12).

### Identifying New Approaches to Optimize Thrombus Characterization for Predicting Early Recanalization and Reperfusion With IV tPA Using Serial CT Angiography (INTERRSeCT) (13)

INTERRSeCT is a prospective multi-center hospital-based cohort of patients treated with intravenous alteplase comparing the rates of early recanalization in 684 patients. Patient eligibility included the following: presentation to the emergency department with symptoms consistent with ischemic stroke 12 h from last known well, age at least 40 years, and a baseline CTA (before alteplase bolus, if given) with evidence of a symptomatic intracranial thrombus. This study compared rates of early recanalization by location of primary intracranial or extracranial artery occlusion. The primary radiological outcome was recanalization, while the primary clinical outcome was functional independence, as measured by the mRS (range, 0 [no symptoms] to 6 [death]) at 90 days. Details about this study have been published elsewhere (13). A subset of 507 patients were included in this study, and the rest of the patients were excluded for being a participant in the PROVE-IT study.

Data from both study cohorts are not publicly available because of data protection laws imposed by University of Calgary Conjoint Health Research Ethics. But datasets might be available from the institutional CHRED for research who meet the criteria for access to confidential data.

### Statistical Analysis

Descriptive statistics were used to compare patients' demographic and clinical characteristics in both training and validation datasets. Similarly, descriptive analyses of patients' characteristics by 90-day functional status (i.e., mRS > 2 vs. mRS ≤ 2) were conducted for each training and validation cohorts. Univariate associations between each categorical/continuous predictor variable and 90-day function outcome were assessed using the chi-square test and Wilcoxon rank-sum tests, respectively. For each cohort, predictors on which missing data were more than 50%, and/or predictors for which the ratio of the levels of categorical features are more than 4:1 in either cohorts were excluded from the analyses. Median imputation method was used to impute missing data in both cohorts. In addition, given the slight imbalance in the distribution of patients by functional outcome, an under-sampling of majority subgroup (mRS ≤ 2 subgroup) was adopted to mitigate the influence of class imbalance on the accuracy of the investigated models in both cohorts.

Functional impairment risk prediction models developed based on support vector machine, random forest, C5.0 decision tree, adaptive boost machine, classification and regression tree, least absolute shrinkage, and selection operator (LASSO) logistic regression, and conventional logistic regression models were trained using under-sampled data from the PROVE-IT study and validated in the INTERRSeCT study. For each cohort, model predictors were scaled to ensure comparability of accuracy across several models. Specifically, predictor scaling was performed in each shuffle in the outer loop and that imputed and scales values were based on the training set both for both cohorts. Two variable selection strategies were adopted for deriving the most accurate risk prediction model for all the ML and regression algorithms, namely; (1) clinical expert knowledge, and (2) automated variable selection. Model predictors were selected based on the knowledge of the literature by two stroke neurologists on our team (BKM, MDH) and their presence in both PROVE-IT and INTERRSeCT cohorts. These predictors included age, NIHSS, treatment received, blood glucose, systolic blood pressure, diastolic blood pressure, hypertension, and diabetes. On the other hand, an automated variable selection method based on rank ordering of the full set of predictors was used to predict 90-day functional outcome. In both approaches, a grid search of the optimal hyper parameter (i.e., hyper parameter tuning) was used to train and derive the most accurate models common to both PROVE-IT and INTERRSeCT cohorts) for each type of model. For LASSO regression, a L1 regularization that shrinks the coefficients effect size of the less important variables toward zero. For random forest, classification and regression trees, and C5.0 decision tree, the optimal number of trees was obtained by grid search while avoiding model overfitting and optimal accuracy. The optimal accuracy of the support vector machine and adaptive boost were obtained by tuning the hyper-parameters using a grid search cross-validation. For the conventional logistic regression, backward elimination was used to determine the most parsimonious model.

Furthermore, predictors in the training data were ranked according to their relative contribution to the prediction of 90-day functional outcomes using a variety of variable importance metrics. For random forest model, the mean decrease in Gini coefficient, which measures how each variable contributes to the homogeneity of the nodes and leaves, was used to rank the variables. Variables with larger Gini index were considered more important (14). For support vector machine and adaptive boosting, the relative importance of each predictor was evaluated based on their unique contribution, as measured by AUC, to the prediction of 90-day functional outcome. On the other hand, the mean decrease in impurity of each surrogate variables at each node was used to evaluate the relative contribution of the predictors in C5.0 and classification and regression trees. For LASSO and the conventional logistic regression models, the magnitude of the standardized logistic regression coefficients was used rank order the predictors according to their importance (15). The absolute values of the importance metrics for all the predictors were scaled to unit norm, in order to ensure comparable rank ordering across all the investigated models (16).

Furthermore, the predictive accuracy of both ML and logistic regression models were assessed using both internal cross-validation (PROVE-IT) and external validation (INTERRSeCT). In the former, the prediction models were trained using data obtained from the PROVE-IT study via a repeated 3-fold cross-validation method. Specifically, the PROVE-IT dataset was randomly split, with two-thirds of the data used for model development and the remaining one-third for internal validation. This process was repeated 500 times by sampling the original data with replacement. In the latter, predictors in the INTERRSeCT data were scaled using parameters from the PROVE-IT study before validating the trained in this cohort.

The predictive performance of each model was examined using sensitivity, specificity, the area under the receiver operating characteristic curve (AUC), and Mathew's correlation coefficient (MCC). MCC measures the strength of association between observed and predicted binary classification. MCC values ranges between −1 and +1; the former represents total disagreement between observed and predicted binary classifications, while the latter represents perfect agreement (i.e., perfect prediction) (17, 18).

Moreover, both brier scores and calibration plots were used to assess the calibration performance of all the models trained and validated in the PROVE-IT and INTERSECT cohorts, respectively (19). In contrast, calibration curves for all the ML and regression-based algorithms based on automated and clinical expert knowledge predictor selection methods were plotted. A perfectly calibrated model should have all the points line on a 45^0^ diagonal line to the x- and y-axes; the greater the deviation of the calibration curve from this diagonal line, the poorer the model calibration. The development and validation of models was checked against the recommendations for reporting in the TRIPOD statement (see Appendix A in Supplementary Material). Statistical significance was evaluated at α = 0.05. All the analyses were conducted using several packages (20–26) in R software v 3.6.1 (27).

## Results

Table 1 describes the demographic and clinical characteristics of patients between PROVE-IT and INTERRSeCT study cohorts. Of the 614 patients in the PROVE-IT study, all the study predictors had no more than 10% missing values, except for imaging. Similarly, of the 507 patients in the INTERRSeCT study, all the study predictors had less than 10% missing values, except for imaging, hemoglobin, partial thromboplastin time, and history of hemoglobin (See Appendix B in Supplementary Material). In addition, history of congestive heart failure, history of heart disease, and smoking had skewed distributions across their categorical levels. These seven variables were excluded from our main analyses. There were no significant differences between both cohorts with respect to patients' demographical and clinical characteristics. Tables 2, 3 describes the demographic and clinical characteristics of patients according to their 90-day functional outcome in PROVE-IT and INTERRSeCT cohorts, respectively. In both cohorts, patients with mRS > 2 tend to be older patients (*p* < 0.01) with more severe stroke (*p* < 0.01), and comorbid hypertension (*p* < 0.01).

**Table 1:** Descriptive characteristics of PROVE-IT and INTERRSeCT study participants.

**Participants' characteristics**	**PROVE-IT**	**INTERRSeCT**	***P*-value**
	**(N_**1**_ = 614)**	**(N_**2**_ = 507)**	
Age (median, IQR)	73 (63–80)	71 (63–79)	0.68
Sex (*n*, % Male)	322 (52.4%)	264 (52.1%)	0.48
Diastolic blood pressure (median, IQR)	82 (74–93)	80 (71–90)	0.62
Systolic blood pressure (median, IQR)	150 (135–170)	147 (131–169)	0.45
Blood glucose (median, IQR)	6.4 (5.6–7.8)	6.5 (5.8–7.9)	0.39
NIHSS (median, IQR)	13 (6–19)	14 (8–19)	0.46
Treatment (*n*, % Intervention)	291 (47.3%)	192 (37.9%)	0.56
History of atrial fibrillation (*n*, % Yes)	184 (29.9%)	161 (31.8%)	0.27
Diabetes (*n*, % No)	511 (83.2%)	421 (83.0%)	0.34
Hypertension (*n*, % Yes)	422 (68.7%)	302 (59.6%)	0.35
International normalize ratio (median, IQR)	1 (1–1.2)	1(1–1.1)	0.60
Creatinine (median, IQR)	81 (67.8–96)	78 (66–93.4)	0.97
Platelet count (median, IQR)	207 (171–253)	195 (57–137)	0.67
Hematocrit (median, IQR)	0.43 (0.39–0.47)	0.41 (0.38–0.44)	0.92

*IQR, Interquartile range; NIHSS, National Institute of Stroke Severity Scale; mRS, modified Rankin Scale*.

**Table 2:** Descriptive characteristics of patients in PROVE-IT study.

**Participants' characteristics**	**mRS > 2**	**mRS ≤ 2**	***P*-value**
	**(N_**1**_ = 249)**	**(N_**2**_ = 365)**	
Age(median, IQR)	77 (69–83)	70 (59–77)	<0.01
Sex (*n*, % Male)	127 (51%)	195 (53.4%)	0.56
Diastolic blood pressure (median, IQR)	85 (77–95)	80 (72–92)	0.09
Systolic blood pressure (median, IQR)	154 (140–172)	150 (130–170)	0.08
Blood glucose (median, IQR)	6.8 (5.8–8.1)	6.2 (5.6–7.5)	<0.01
NIHSS (median, IQR)	17 (11–22)	9 (5–15)	<0.01
Treatment (*n*, % Intervention)	128 (51.1%)	163(44.7%)	0.11
History of atrial fibrillation (*n*, % Yes)	92 (36.9%)	92 (25.2%)	0.002
Diabetes (*n*, % No)	202 (81.1%)	309 (84.7%)	0.25
Hypertension (*n*, % Yes)	190 (76.3%)	232 (63.5%)	<0.01
International normalized ratio (median, IQR)	1.09 (1.0–1.20)	1.0 (1.0–1.1)	<0.01
Creatinine (median, IQR)	83 (65–101)	80 (69–91)	0.23
Platelet count (median, IQR)	210 (169–257)	206 (172–249)	0.83
Hematocrit (median, IQR)	0.42 (0.38–0.47)	0.43 (0.41–0.48)	0.03

*IQR, Interquartile Range; NIHSS, National Institute of Health Stroke Severity Scale; mRS, Modified Rankin scale*.

**Table 3:** Descriptive characteristics of patients in the INTERRSeCT Study.

**Participants' characteristics**	**mRS > 2**	**mRS ≤ 2**	***P*-value**
	**(N_**1**_ = 239)**	**(N_**2**_ = 268)**	
Age (median, IQR)	77 (68–83)	68 (59–75)	<0.01
Sex (*n*, %Male)	112 (46.8%)	152 (56.7%)	0.03
Diastolic blood pressure (median, IQR)	80 (70–90)	81 (72–90)	0.71
Systolic blood pressure (median, IQR)	150 (134–171)	145 (130–162)	0.02
Blood glucose (median, IQR)	6.6 (5.9–8.1)	6.4 (5.80–7.55)	0.07
NIHSS (median, IQR)	17 (12–20)	10 (6–17)	<0.01
Treatment (*n*, % Intervention)	86 (35.9%)	106 (39.5)	0.40
History of atrial fibrillation (*n*, % Yes)	88 (36.8%)	73 (27.2%)	0.02
Diabetes (*n*, % No)	192 (80.3%)	229 (85.4%)	0.12
Hypertension (*n*, % Yes)	154 (64.4%)	148 (55%)	0.04
Creatinine (median, IQR)	78 (65–96)	78 (66–91.1)	0.72
International normalized ratio (median, IQR)	1.02 (1.0–1.10)	1.0 (1.0–1.10)	0.09
Platelet Count (median, IQR)	93 (57–142)	97 (55.8–130.3)	0.88
Hematocrit (median, IQR)	0.4 (0.37–0.43)	0.42 (0.39–0.44)	<0.01

*IQR, Interquartile Range; NIHSS, National Institute of Health Stroke Severity Scale; mRS, Modified Rankin scale*.

**Figure 1:**
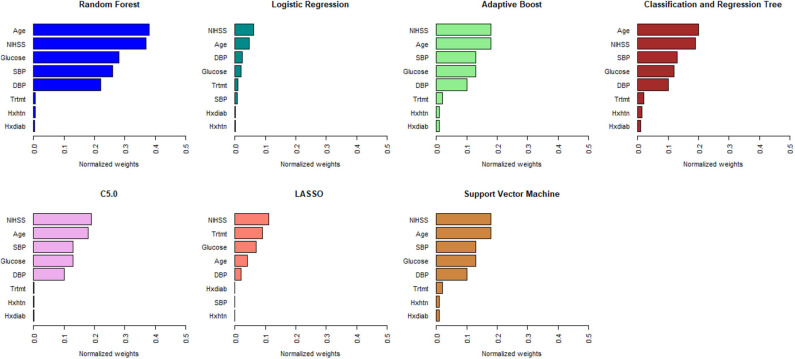
Rank ordering of machine learning and regression model predictors (selected based on clinical expert knowledge). NIHSS, National Institute of Health and Stroke Scale; SBP, Systolic Blood Pressure; DBP, Diastolic Blood Pressure; Trtmt, Treatment; Hxdiab, History of Diabetes; Hxhtn, History of Hypertension; LASSO, Least Absolute Shrinkage Selection Operator.

**Figure 2:**
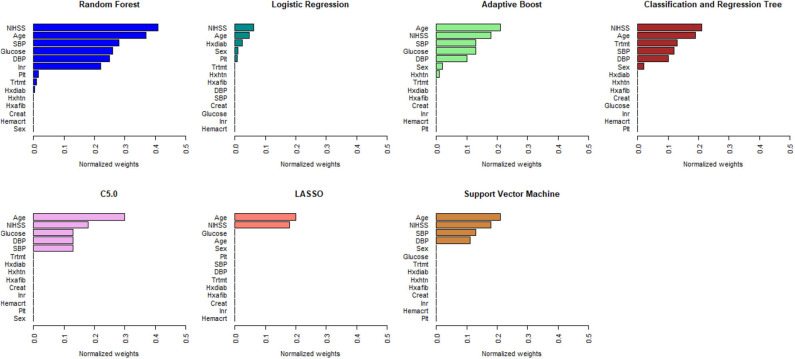
Rank ordering of machine learning and regression-based model predictors (automated variable selection). NIHSS, National Institute of Health and Stroke Scale; SBP, Systolic Blood Pressure; DBP, Diastolic Blood Pressure; Trtmt, Treatment; Hxdiab, History of Diabetes; Hxhtn, History of Hypertension; Hxafib, History of Atrial Fibrillation; Plt, Partial Thromboplastin Time; Inr, International Normalize Ratio; Creat, Creatinine; Hemacrt, Hematocrit; LASSO, Least Absolute Shrinkage Selection Operator.

Figures 1, 2 describe the relative importance of the predictor variables with respect to the prediction of 90-day functional outcomes in ischemic stroke patients for ML and logistic regression models in imputed PROVE-IT data. Age and NIHSS were ranked as the two most important predictors of 90-day functional outcomes for almost all the models, regardless of the predictor selection strategy. But there are variations in the rank ordering other less important variables across all the investigated models.

Table 4 describes the predictive accuracy of investigated models in the PROVE-IT data when the predictors were selected based on automated variable selection and clinical expert knowledge. There were negligible differences in the accuracy of ML and regression-based models, regardless of the variable selection method adopted. For example, for models trained using predictors derived from clinical expert knowledge, the average AUC and MCC for LASSO logistic regression were 0.71 (95%CI = [0.53, 0.71]) and 0.43(95%CI = [0.32, 0.55]); whereas the average AUC and MCC for random forest were 0.67 (95%CI = [0.61, 0.73]) and 0.34 (95%CI = [0.22, 0.46]). Similar patterns were observed for both sensitivity and specificity values across the models. Moreover, when these models were validated externally in the INTERRSeCT cohort (Table 5), similar patterns of negligible differences in the AUCs and MCCs of ML and logistic regression algorithms were reported, regardless of the variable selection strategies adopted.

**Table 4:** Predictive accuracy (95%CI) of regression and ML models in PROVE-IT data (internal validation).

**Predictive accuracy metric**	**RF**	**SVM**	**C5.0**	**ABM**	**CART**	**LR**	**LASSO**
**Automated variable selection**							
Sensitivity	0.67 (0.57–0.77)	0.62 (0.51–0.72)	0.66 (0.56–0.76)	0.74 (0.64–0.83)	0.76 (0.66–0.84)	0.73 (0.63–0.83)	0.73 (0.63–0.82)
Specificity	0.58 (0.47–0.68)	0.63 (0.52–0.73)	0.71 (0.61–0.80)	0.60 (0.50–0.71)	0.54 (0.43–0.64)	0.70 (0.60–0.79)	0.70 (0.60–0.79)
AUC	0.63 (0.56–0.70)	0.62 (0.55–0.70)	0.69 (0.62–0.76)	0.67 (0.60–0.74)	0.65 (0.58–0.72)	0.72 (0.65–0.78)	0.72 (0.65–0.79)
MCC	0.26 (0.11–0.39)	0.25 (0.11–0.37)	0.38 (0.24–0.51)	0.35 (0.21–0.49)	0.31 (0.16–0.44)	0.43 (0.30–0.56)	0.43 (0.30–0.56)
Brier score	0.34 (0.30–0.45)	0.34 (0.30–0.45)	0.28 (0.25–0.38)	0.30 (0.21–0.48)	0.32 (0.28–0.42)	0.26 (0.21–0.35)	0.26 (0.21–0.35)
**Clinical expert knowledge**							
Sensitivity	0.64 (0.55–0.72)	0.61 (0.51–0.71)	0.68 (0.59–0.76)	0.66 (0.57–0.75)	0.62 (0.53–0.71)	0.62 (0.53–0.71)	0.62 (0.53–0.71)
Specificity	0.70 (0.60–0.79)	0.77 (0.67–0.84)	0.69 (0.59–0.78)	0.74 (0.64–0.82)	0.71 (0.69–0.79)	0.80 (0.72–0.87)	0.80 (0.72–0.87)
AUC	0.67(0.61–0.73)	0.69 (0.63–0.75)	0.69 (0.63–0.75)	0.70 (0.64–0.76)	0.67 (0.61–0.73)	0.71(0.66–0.77)	0.71 (0.66–0.77)
MCC	0.34 (0.22–0.46)	0.38 (0.26–0.50)	0.37 (0.24–0.49)	0.40 (0.28–0.52)	0.33 (0.21–0.47)	0.43 (0.32–0.55)	0.43 (0.32–0.55)
Brier score	0.33 (0.27–0.39)	0.31 (0.26–0.38)	0.31 (0.25–0.37)	0.30 (0.24–0.36)	0.34 (0.28–0.39)	0.29 (0.24–0.35)	0.29 (0.24–0.35)

*95%CI, 95% Confidence Interval; AUC, Area under the receiver operating characteristic curve; RF, Random Forest; SVM, Support Vector Machine; C5.0, C5.0 Decision Tree; ABM, Adaptive Boost Machine; CART, Classification and Regression Tree; LR, Logistic Regression; LASSO, Least Absolute Shrinkage and Selection Operation; MCC, Matthews Correlation Coefficient*.

**Table 5:** Predictive accuracy (95%CI) of regression and ML models in INTERRSeCT data (external validation).

**Predictive accuracy metric**	**RF**	**SVM**	**C5.0**	**ABM**	**CART**	**LR**	**LASSO**
**Automated variable selection**							
Sensitivity	0.70 (0.64–0.76)	0.65 (0.59–0.71)	0.76 (0.70–0.81)	0.68 (0.61–0.74)	0.77 (0.71–0.82)	0.83 (0.78–0.88)	0.71 (0.60–0.79)
Specificity	0.71 (0.65–0.77)	0.77 (0.71–0.82)	0.58 (0.51–0.65)	0.70 (0.60–0.79)	0.60 (0.54–0.67)	0.56 (0.49–0.62)	0.67 (0.61–0.73)
AUC	0.70 (0.66–0.75)	0.71 (0.65–0.75)	0.66 (0.63–0.72)	0.67 (0.65–0.73)	0.69 (0.64–0.73)	0.69 (0.65–0.73)	0.67 (0.60–0.73)
MCC	0.41 (0.29–0.54)	0.42 (0.29–0.53)	0.34 (0.15–0.44)	0.38 (0.16–0.43)	0.38 (0.14–0.41)	0.40 (0.29–0.52)	0.39 (0.20–0.50)
Brier score	0.32 (0.28–0.41)	0.32 (0.29–0.42)	0.32 (0.28–0.41)	0.32 (0.29–0.42)	0.33 (0.30–0.43)	0.30 (0.27–0.39)	0.30 (0.27–0.39)
**Clinical expert knowledge**							
Sensitivity	0.67 (0.61–0.73)	0.67 (0.61–0.73)	0.67 (0.60–0.73)	0.56 (0.49–0.62)	0.66 (0.60–0.72)	0.81 (0.75–0.85)	0.71 (0.65–0.77)
Specificity	0.71 (0.65–0.76)	0.72 (0.66–0.78)	0.75 (0.69–0.80)	0.78 (0.73–0.83)	0.69 (0.62–0.75)	0.60 (0.54–0.66)	0.69 (0.63–0.75)
AUC	0.66 (0.58–0.73)	0.67 (0.65–0.74)	0.63 (0.58–0.67)	0.68 (0.63–0.72)	0.67 (0.63–0.72)	0.68 (0.65–0.72)	0.69 (0.65–0.73)
MCC	0.38 (0.22–0.55)	0.39 (0.27–0.55)	0.42 (0.28–0.51)	0.35 (0.21–0.53)	0.35 (0.21–0.54)	0.41 (0.29–0.55)	0.40 (0.29–0.53)
Brier score	0.31 (0.25–0.37)	0.26 (0.22–0.38)	0.35 (0.27–0.36)	0.25 (0.21–0.32)	0.25 (0.21–0.38)	0.27(0.25–0.36)	0.27(0.24–0.35)

*95%CI, 95% Confidence Interval; AUC, Area under the receiver operating characteristic curve; RF, Random Forest; SVM, Support Vector Machine; C5.0, C5.0 Decision Tree; ABM, Adaptive Boost Machine; CART, Classification and Regression Tree; LR, Logistic Regression; LASSO, Least Absolute Shrinkage and Selection Operation; MCC, Matthews Correlation Coefficient*.

**Figure 3:**
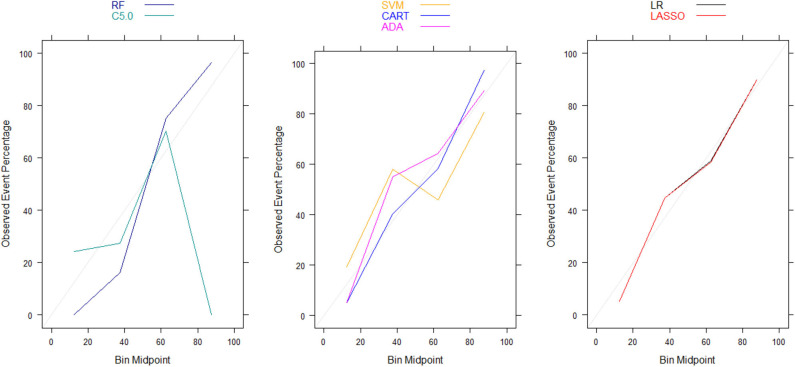
Calibration plots for externally validated ML and regression-based models based on predictors derived via clinical expert knowledge. RF, Random Forest; SVM, Support Vector Machine; C5.0, C5.0 Decision Tree; ADA, Adaptive Boost Machine; CART, Classification and Regression Tree; LR, Logistic Regression; LASSO, Least Absolute Shrinkage and Selection Operation.

**Figure 4:**
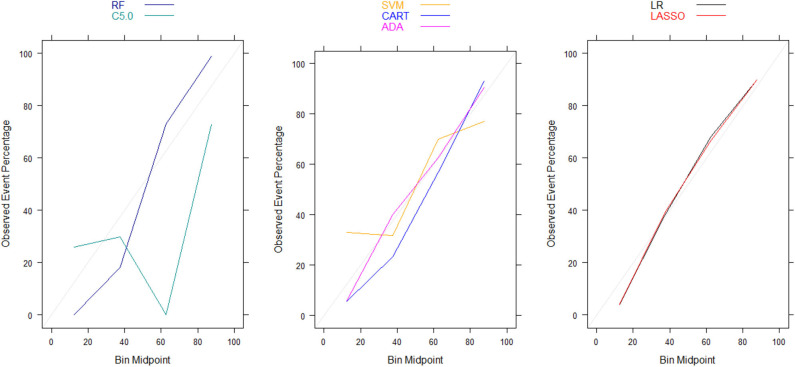
Calibration plots for externally validated ML and regression-based models based on automated predictor selection. RF, Random Forest; SVM, Support Vector Machine; C5.0, C5.0 Decision Tree; ADA, Adaptive Boost Machine; CART, Classification and Regression Tree; LR, Logistic Regression; LASSO, Least Absolute Shrinkage and Selection Operation.

Moreover, there were no significant differences in Brier scores for ML and regression-based models across both training and validation cohorts, regardless of the variable selection strategy adopted (Tables 4, 5). But graphical examination of calibration for LASSO logistic and conventional logistic regression models showed that both models had fairly good calibration in high-risk individuals but they slightly under-estimated or over-estimated the risk of poor functional outcome (mRS > 3) in low risk in individuals. In contrast, the calibration slopes for classification and regression tree exhibited moderate departures from the diagonal line, suggesting moderately poor calibration. But the random forest, C5.0 decision tree, support vector machine and adaptive boosting significantly over-estimated and/or under-estimated the probability of poor functional outcomes in low risk and high risk individuals (See Figures 3, 4).

## Discussion

This study examined the predictive accuracy of regression-based and ML models for predicting functional outcomes in stroke patients. Our analyses revealed that ML algorithms and logistic regression models had comparable predictive accuracy when validated internally and externally. Our findings buttress current evidence from other published studies (28–33) that already showed that the logistic regression and ML algorithms had comparable predictive accuracy in empirical clinical studies. A recently published systematic review found no evidence of the superior predictive performance of ML models over logistic regression models in clinical studies (32). Also, Van Os et al. (33) also explored the use of ML algorithms for predicting 90-day functional outcomes in MR CLEAN registry, a Dutch database of stroke patients who received endovascular treatment, and concluded that ML algorithms did not exhibit superior predictive accuracy over logistic regression models. These studies, while similar to ours in their use of feature selection predictor selection, relied on a relatively larger sample size than ours (*N* > 1,000) but lacked validation of their prediction algorithms in external cohorts.

Moreover, our calibration plots revealed that logistic regression models had good calibration but ML algorithms had poorer calibration despite having comparable Brier scores. In fact, the majority of the ML algorithms had either overfitting or under-fitting problems in correctly predicting patients' functional outcomes despite having Brier scores and AUCs that are comparable to those in logistic regression models. This highlights the inherent limitation of interpreting Brier score, which is a composite measure of both discrimination and calibration, in terms of calibration alone (19). Therefore, our conclusions about the calibration of ML algorithms are based on the calibrations curves rather than Brier scores. This is consistent with recommendations by Rufibach (19) who cautioned against erroneous interpretation of the low Brier score as indicative of good calibration. On the other hand, our evaluation of the relative importance of the predictor variables in risk prediction models based on ML and logistic regression revealed that age and stroke severity (measured by NIHSS) were the most important predictors of 90-day functional outcome that are common across all the models. This is consistent with findings from existing prognostic risk scores for predicting functional outcomes in ischemic stroke (3, 34–36), many of which have identified stroke severity (measured by NIHSS) and age as most important predictors of 90-day functional outcomes in ischemic stroke patients.

A unique strength of this study is the examination of both the discrimination and calibration of the investigated ML and regression-based risk prediction models. Unlike most clinical prediction studies that lack external validation of their findings, the external validation of these ML and regression-based functional impairment risk prediction models developed in another observational stroke registry is also a unique feature of this study. Despite these strengths, this study is not without its limitations, which might have influenced our study conclusions. First, this study focused primarily on regression and ML models for predicting binary outcomes to derive more accurate estimates of functional impairment risk. Our conclusions are based on empirical analysis of observational cohorts of acute stroke patients. This might limit the generalizability of findings to other populations. For example, both PROVE-IT and INTERRSeCT are small-sampled hospital cohorts and study predictors are those collected in acute care settings. Other important post-acute care risk factors, such as social support, imaging, and stroke rehabilitation, which are known to be predictive of 90-day functional impairment risk, were either not at our disposal or had significant missing data (37, 38). Future research will examine the robustness of our conclusions in large observational cohorts and through the use of computer simulations to study the performance of these models under a variety of different data analytic conditions. Second, our choice of PROVE-IT study cohort as the training cohort was driven by our initial access to this multicenter prospective observational study led by members of our team (BKM, MDH) and the relatively smaller rate of missing observations in PROVE-IT. It is possible that our conclusions might be different if INTERRSeCT study was used to train and develop the models while PROVE-IT study is used for external validation. Third, we have adopted mRS02 as the binary cut off point for good outcomes. It is possible that our study conclusions might be sensitive to others definitions of the good outcome functional outcomes (e.g., mRS01, or mRS03). Finally, median imputation method to impute missing observations in both cohorts was used. Future research will use sensitivity analyses will examine the robustness of our study conclusions to different types of imputation methods such as the multiple imputation chain equation methods.

In conclusion, ML and regression-based models have comparable predictive accuracy in predicting functional outcomes in stroke patients. We recommend that the choice between among these classes of models should be guided by important considerations as study design characteristics, data quality, and its utility in clinical settings.

## Data Availability Statement

The data analyzed in this study is subject to the following licenses/restrictions: Data from both study cohorts are not publicly available because of data protection laws imposed by University of Calgary Conjoint Health Research Ethics (UCalgary CHREB). But datasets might be available from the UCalgary CHREB for researchers who meet the criteria for access to confidential data. Requests to access these datasets should be directed to docbijoymenon@gmail.com.

## Ethics Statement

The studies involving human participants were reviewed and approved by University of Calgary Conjoint Health Research Ethics Board (REB14-2012 and REB14-2015). The patients/participants provided their written informed consent to participate in this study.

## Author Contributions

All authors listed have made a substantial, direct and intellectual contribution to the work, and approved it for publication.

## Conflict of Interest

The authors declare that the research was conducted in the absence of any commercial or financial relationships that could be construed as a potential conflict of interest.
