# Supplementary material for: Functional Outcome Prediction in Ischemic Stroke: A Comparison of Machine Learning Algorithms and Regression Models
Source: Front Neurol. 2020 Aug 25;11:889. doi: 10.3389/fneur.2020.00889 (PMC7479334; doi:10.3389/fneur.2020.00889)
Supplement: Supplementary file 1 [file Data_Sheet_1.PDF]

TRIPOD Checklist: Prediction Model Development and Validation

Section/Topic	Item		Checklist Item	Page
**Title and abstract**				
Title	1	D;V	Identify the study as developing and/or validating a multivariable prediction model, the target population, and the outcome to be predicted.<br><br>The main objective for this manuscript is not to develop and validate a clinical risk prediction model for use in clinical settings; therefore the title does not follow this format.<br><br>The manuscript does however involve the development and internal validation of logistic regression and machine learning algorithms risk prediction model, and we report on the guidelines below for this process, where relevant.	1
Abstract	2	D;V	Provide a summary of objectives, study design, setting, participants, sample size, predictors, outcome, statistical analysis, results, and conclusions.	3
**Introduction**				
Background and objectives	3a	D;V	Explain the medical context (including whether diagnostic or prognostic) and rationale for developing or validating the multivariable prediction model, including references to existing models.	4
	3b	D;V	Specify the objectives, including whether the study describes the development or validation of the model or both.<br><br>Our study objectives did not explicitly focus on model development and validation but on the comparison on the accuracy of logistic regression and machine learning models.	5
**Methods**				
Source of data	4a	D;V	Describe the study design or source of data (e.g., randomized trial, cohort, or registry data), separately for the development and validation data sets, if applicable.	6
	4b	D;V	Specify the key study dates, including start of accrual; end of accrual; and, if applicable, end of follow-up.	NA
Participants	5a	D;V	Specify key elements of the study setting (e.g., primary care, secondary care, general population) including number and location of centres.	6
	5b	D;V	Describe eligibility criteria for participants.	6
	5c	D;V	Give details of treatments received, if relevant.	6
Outcome	6a	D;V	Clearly define the outcome that is predicted by the prediction model, including how and when assessed.	6-7
	6b	D;V	Report any actions to blind assessment of the outcome to be predicted.	NA
Predictors	7a	D;V	Clearly define all predictors used in developing or validating the multivariable prediction model, including how and when they were measured.	8
	7b	D;V	Report any actions to blind assessment of predictors for the outcome and other predictors.	NA
Sample size	8	D;V	Explain how the study size was arrived at.<br><br>The PROVE-IT sample included all patients in the cohort study who were not part of the INTERRSeCT study and vice versa	6
Missing data	9	D;V	Describe how missing data were handled (e.g., complete-case analysis, single imputation, multiple imputation) with details of any imputation method.	7
Statistical analysis methods	10a	D	Describe how predictors were handled in the analyses.	8
	10b	D	Specify type of model, all model-building procedures (including any predictor selection), and method for internal validation.	8-9
	10c	V	For validation, describe how the predictions were calculated.	9
	10d	D;V	Specify all measures used to assess model performance and, if relevant, to compare multiple models.	9-10
	10e	V	Describe any model updating (e.g., recalibration) arising from the validation, if done.	NA
Risk groups	11	D;V	Provide details on how risk groups were created, if done.	NA
Development vs. validation	12	V	For validation, identify any differences from the development data in setting, eligibility criteria, outcome, and predictors.<br><br>Both training and validation datasets were compared in terms of their characteristics	7, 22
**Results**				
Participants	13a	D;V	Describe the flow of participants through the study, including the number of participants with and without the outcome and, if applicable, a summary of the follow-up time. A diagram may be helpful.	6
	13b	D;V	Describe the characteristics of the participants (basic demographics, clinical features, available predictors), including the number of participants for predictors and outcome.	7, 22
	13c	V	For validation, show a comparison with the development data of the distribution of important variables (demographics, predictors and outcome).	7, 10
Model development	14a	D	Specify the number of participants and outcome events in each analysis.	10, 23-24
	14b	D	If done, report the unadjusted association between each candidate predictor and outcome.	10. 23-24
Model specification	15a	D	Present the full prediction model to allow predictions for individuals (i.e., all regression coefficients, and model intercept or baseline survival at a given time point)	NA

TRIPOD Checklist: Prediction Model Development and Validation

	15b	D	Explain how to the use the prediction model.	NA
Model performance	16	D;V	Report performance measures (with CIs) for the prediction model.	25-26
Model-updating	17	V	If done, report the results from any model updating (i.e., model specification, model performance).	NA
**Discussion**<br><br>**The discussion section was structured differently because the aim of this paper was not the development and validation of a model to be used in practice**				
Limitations	18	D;V	Discuss any limitations of the study (such as nonrepresentative sample, few events per predictor, missing data).	14
Interpretation	19a	V	For validation, discuss the results with reference to performance in the development data, and any other validation data.	11, 14
	19b	D;V	Give an overall interpretation of the results, considering objectives, limitations, results from similar studies, and other relevant evidence.	12-15
Implications	20	D;V	Discuss the potential clinical use of the model and implications for future research.	14
**Other information**				
Supplementary information	21	D;V	Provide information about the availability of supplementary resources, such as study protocol, Web calculator, and data sets.	10
Funding	22	D;V	Give the source of funding and the role of the funders for the present study.	16

*Items relevant only to the development of a prediction model are denoted by D, items relating solely to a validation of a prediction model are denoted by V, and items relating to both are denoted D;V.
