# Supplementary material for: Functional Outcome Prediction in Ischemic Stroke: A Comparison of Machine Learning Algorithms and Regression Models
Source: Front Neurol. 2020 Aug 25;11:889. doi: 10.3389/fneur.2020.00889 (PMC7479334; doi:10.3389/fneur.2020.00889)
Supplement: Supplementary file 2 [file Table_1.DOCX]

Table A1: Distribution of Missing data in PROVE-IT & INTERRSeCT cohorts

*NB: IQR =Interquartile Range; NIHSS = National Institute of Health Stroke Severity Scale; mRS = modified Rankin Scale

| Participants’ Characteristics | INTERRSeCT | PROVE-IT |
| --- | --- | --- |
| Age(median, IQR) | 20 (3.9%) | 17 (2.76%) |
| Sex(n, %Male) | 20 (3.9%) | 18 (2.9%) |
| Diastolic blood pressure(median, IQR) | 23 (4.5%) | 26 (4.23%) |
| Systolic blood pressure(median, IQR) | 23 (4.5%) | 25 (4.07%) |
| Blood glucose(median, IQR) | 21 (4.1%) | 26 (4.23%) |
| NIHSS (median, IQR) | 21 (4.1%) | 18 (2.93%) |
| History of Congestive Heart Failure (n, % Yes) | 20 (3.9%) | 20 (3.26 %) |
| Treatment (n, % Intervention) | 20 (3.9%) | 18 (2.93%) |
| History of Atrial Fibrillation(n, %Yes) | 20 (3.9%) | 21 (3.42%) |
| Smoking (n, % Yes) | 29 (5.7%) | 35 (5.7%) |
| Diabetes (n, %No) | 20 (3.9%) | 22 (3.58%) |
| Hypertension (n, % Yes)  History of Heart Diseases (n, % Yes)  Imaging (n, % Left Side Affected)  History of Hemoglobin (median, IQR)  Creatinine (median, IQR)  Heart Rate (median, IQR)  International Normalize Ratio (median, IQR)  Platelet Count (median, IQR)  Partial Thromboplastin Time (median, IQR)  Hematocrit (median, IQR)  Modified rankin scale | 20 (3.9%)  20 (3.9%)  286 (56.4%)  298 (58.7%)  20 (3.9%)  49 (9.7%)  22 (4.3%)  21 (4.1%)  26 (5.1%)  20 (3.9%)  20 (3.9%) | 20 (3.26%)  20 (3.26 %)  71 (11.6%)  24 (3.91%)  28 (4.56%)  50 (8.14%)  30 (4.89%)  26 (4.23%)  53 (8.63%)  26 (4.23%)  28 (4.56%) |
